# Feeling Healthy? A Survey of Physical and Psychological Wellbeing of Students from Seven Universities in the UK

**DOI:** 10.3390/ijerph8051308

**Published:** 2011-04-27

**Authors:** Walid El Ansari, Christiane Stock, the UK Student Health Group, Sherrill Snelgrove, Xiaoling Hu, Sian Parke, Shân Davies, Jill John, Hamed Adetunji, Mary Stoate, Pat Deeny, Ceri Phillips, Andi Mabhala

**Affiliations:** 1 Faculty of of Applied Sciences, University of Gloucestershire, Oxstalls Campus, Oxstalls Lane, Gloucester GL2 9HW, UK; 2 Unit for Health Promotion Research, Institute of Public Health, University of Southern Denmark, Niels Bohrs Vej 9-10, 6700 Esbjerg, Denmark; E-Mail: cstock@health.sdu.dk; 3 School of Human and Health Sciences, Swansea University, Singleton Park, Swansea SA2 8PP, Wales, UK; E-Mails: s.r.snelgrove@swansea.ac.uk (S.S.); s.parke@swansea.ac.uk (S.P.); shan.davies@swansea.ac.uk (S.D.); j.e.john@swansea.ac.uk (J.J.); c.j.phillips@swansea.ac.uk (C.P.); 4 Business School, University of Gloucestershire, Cheltenham, Gloucestershire GL50 2RH, UK; E-Mail: xhu@glos.ac.uk; 5 School of Health & Social Care, Oxford Brookes University, Marston, Oxford OX3 0FL, UK; E-Mail: hadetunji@brookes.ac.uk; 6 School of Science, Society and Management, Bath Spa University, Newton St. Loe, Bath BA2 9BN, UK; E-Mail: m.stoate@bathspa.ac.uk; 7 Institute of Nursing Research, School of Nursing, University of Ulster, Londonderry, Northern Ireland BT48 7Jl, UK; E-Mail: pg.deeny@ulster.ac.uk; 8 Faculty of Health and Social Care, University of Chester, Castle Drive, Chester CH1 4BJ, UK; E-Mail: a.mabhala@chester.ac.uk

**Keywords:** university students, physical health, psychological wellbeing, social support, psychosomatic, burdens and stressors, gender

## Abstract

University students’ physical and psychological health and wellbeing are important and comprise many variables. This study assessed perceived health status in addition to a range of physical and psychological wellbeing indicators of 3,706 undergraduate students from seven universities in England, Wales and Northern Ireland. We compared differences in these variables across males and females, and across the participating universities. The data was collected in 2007–2008. A self-administered questionnaire assessed socio-demographic information (e.g., gender, age), self-reported physical and psychological health data, as well as questions on health awareness, health service use, social support, burdens and stressors and university study related questions. While females generally reported more health problems and psychological burdens, male students felt that they received/had fewer persons to depend on for social support. The comparisons of health and wellbeing variables across the different universities suggested some evidence of ‘clustering’ of the variables under study, whereby favourable situations would be exhibited by a cluster of the variables that is encountered at some universities; and conversely, the clustering of less favourable variables as exhibited at other universities. We conclude that the level of health complaints and psychological problems/burdens is relatively high and calls for increased awareness of university administrators, leaders and policy makers to the health and well-being needs of their students. The observed clustering effects also indicated the need for local (university-specific) health and wellbeing profiles as basis and guidance for relevant health promotion programmes at universities.

## 1. Introduction

University students represent the future of families, communities, and countries. They also face the stresses of achieving success in their academic goals despite the financial constraints that many students report [1]. University is a period of increased responsibility for choices and healthy practices [2]. Lifestyle characterised by unhealthy practices might not show an effect on health in the short and interim terms [3], but such ‘habits’ could persist into middle and old age to inflict health hazards later in life. Indeed it is challenging for adults to modify the potentially harmful habits instigated in their youth [4]. This is particularly relevant when unhealthy behaviours cluster together (possibly leading to co-morbidities later in life). For instance, nearly 65% of women aged 18–22 enrolled full-time at an urban university in the USA had two or more unhealthy behaviours [5]. Further, the average weight gain during the first semester of college for first-time freshmen was 1.3–3.1 kg [6,7].

Indeed studies have suggested that university students’ physical and psychological/mental health and wellbeing are important [1,8-11] and comprise a wide range of aspects. Some research showed that university students reported more health complaints than their working peers [12,13], but did not appear to seek help for these problems [14]. A high prevalence of such complaints has also been documented in university students from different European countries (e.g., [8,15]), which included nervousness, headache and back ache or neck/shoulder ache, but comparative data from the UK are lacking.

Poor ratings of one’s perceived health, along with self-reported symptoms are often mirrored in unfavourable ratings of one’s quality of life. Not surprisingly, students in Sweden reported lower perceived quality of life when compared with their working peers [13], and similar findings have been reported in the UK [12]. Overall, it could be argued that psychosomatic health complaints and impairments in quality of life observed in university students might be associated with study related burdens and stressors. Few studies have examined the perceived burdens of university students, such as the challenges of achieving good grades and competition, career and future achievements, the many demands and deadlines of course works and academic assessments, as well as the financial and health-related burdens [16], and their impact on health [17]. Recent research concluded that perceived burdens were positively associated with higher depression scores among students, not only by mediation through perceived stress but also directly [18].

Although university students are confronted with potential stressors as outlined above, it has also been reported that the majority of students have a high level of social support [19]. Certainly, social support has been viewed as a potential buffer against harmful effects of psychological stress [20] and has therefore the potential of being a resource for health in this population group.

### 

#### Aim of the Study

Although several studies have highlighted different aspects of student health and well-being, little research has included different indicators of student health, quality of life and study-related burdens, in addition to focussing on resources like social support. Therefore, the current study investigated perceived health status, a range of physical and mental/psychological wellbeing variables, and as well as social support of students from seven universities in England, Wales and Northern Ireland. The four specific objectives were to:

- Describe the socio-demographic characteristics of students (e.g., age, gender, marital status and children, living arrangements, financial sufficiency, and the importance of faith);
- Assess the prevalences of a variety of physical health and wellbeing variables (e.g., subjective general health, health awareness, health service use, and physical health problems/strains);
- Assess the prevalences of a variety of psychological health and mental wellbeing variables (e.g., quality of life; social support, satisfaction with social support, perceived burdens and psychosomatic health problems/strains); and,
- Compare data from the participating sites in relation to the self-reported physical health as well as the mental/psychological health and wellbeing of their students.

## 2. Methods

### 2.1. Sample and Data Collection Procedures

Data used in the present analysis was collected as part of the General Student Health Survey [1,19,21]. Cross sectional epidemiological studies are particularly useful for establishing prevalences and identifying underlying risk factors [22]. The UK data used in this analysis was collected at the same time from all participating universities in 2007–2008. For universities in the UK, the typical academic year usually starts towards the end of September and lasts until July the following year. The UK data comprised 3,706 students (765♂ and 2,699♀; mean age 24.9 years, SD 8.6) at seven universities in three countries of the UK: England (University of Gloucestershire, Bath Spa University, Oxford Brookes University, University of Chester, Plymouth University); Wales (Swansea University); and the Republic of Northern Ireland (University of Ulster). The sites were chosen on the basis of research interests, existing contacts and history of successful previous collaboration. Ethical approval was provided by the participating institutions. Towards the middle of the term/semester, self-administered questionnaires were distributed to students attending regular classes of randomly selected courses at the universities during the last 5–10 minutes of their lectures. No incentives were provided, each questionnaire had an information sheet outlining the research objectives, and student participation was voluntary and anonymous. Data were confidential and protected at all stages of the study. A representative sample of students was sought at all participating universities, and students were informed that by completing the questionnaire, they agreed to participate in the study. All data were computer entered at one site using the software Teleform®, thus maximising the quality assurance and minimising errors of data entry. Similar to other student health [1,19] and educational satisfaction [23] surveys, based on the number of returned questionnaires, the response rates were ≈80%.

### 2.2. Health and Wellbeing Questionnaire: Physical and Psychological Health

The study was a general student health and wellbeing survey similar to studies of student health implemented in several countries [19,21]. It included socio-demographic information (e.g., gender, age), self-reported health data, as well as questions on health awareness, health service use, social support, burdens and stressors and university study related questions.

*General health and health awareness* (2 items): these inquired about general health and were adopted from The American College Health Association [9]. Students rated their current general health by the question: “How would you describe your general health?” with a five-point response scale (1 = ‘excellent’ to 5 = ‘poor’, later recoded to 3 categories). A related item [8] asked students about their general awareness of their health: “To what extent do you keep an eye on your health?”, with a four-point response scale (1 = ‘not at all’ and 4 = ‘very much’, later recoded to 2 categories).

*Health service use and severe illnesses* (2 items): participants were asked: “Have you seen a medical practitioner (excluding a dentist) in the past 6 months?”, and “During the past 12 months, have you been so ill that you had to stay in bed?”, both with dichotomous ‘yes’/‘no’ response [8]. Participants who answered ‘yes’ to the former item were then asked about the number of times they had seen a medical practitioner (later recoded to 3 categories: ‘1–2 times’, ‘3–4 times’ or ‘≥5 times’).

*Health problems, strains and psychosomatic symptoms* (22 items): students rated 22 symptoms measuring a range of health complaints as adopted from previous studies [8,11,15,24,25]. Sample items included stomach trouble/heartburn, back pain, rapid heart beats/circulatory problem/dizziness, headaches, sleep disorder/insomnia, concentration difficulties, neck and shoulder pain, and depressive mood. Respondents rated the question: “How often have you had these complaints during the past 12 months?” on a four-point response scale (1 = ‘never’; 4 = ‘very often’). The scale had a Cronbach’s alpha of 0.88. For the purpose of the analysis undertaken in this paper, we recoded ‘sometimes’ and ‘very often’ into one category.

*Quality of one’s life* (1 item): measured by the question: “If you consider the quality of your life: How did things go for you in the last four weeks?”. The item was based on the COOP/WONCA charts [26] with the 5 response categories ranging from ‘1 = very badly’ to ‘5 = very well’. This variable was further recoded into two new categories.

*Social support and satisfaction with social support* (2 items): measured by the modified Sarason’s Social Support Questionnaire [27], using two questions: “How many people do you know—including your family and friends—support you whenever you feel down?”. The numerical response was recoded into ‘low’ (1 person), ‘medium’ (2–3 persons) or ‘high’ (>3 persons) social support. Satisfaction with social support was measured by the item: “Are you on the whole satisfied with the support you get in such situations?” using a 5 point Likert scale (1 = ‘very satisfied’, 5 = ‘very dissatisfied’, later recoded into 3 categories).

*Perceived burdens/Life stressors* (18 items): these appraised a range of burdens as perceived by the students by assessing burdens associated with course work and exams, relationships to peers and parents, isolation, financial situation, and expectations regarding the future generally and future job prospects, adopted from published studies [8,15]. The scale had a Cronbach’s alpha of 0.87. Items were introduced with the question: “To what extent do you feel burdened in the following areas?”, with the 6 response categories ranging from ‘not at all’ to ‘very strongly’, subsequently recoded into 2 categories.

### 2.3. Statistical Analysis

SPSS 14.0 (SPSS Inc. Chicago, IL) was used to calculate frequencies and proportions and to conduct the statistical analyses. Frequencies are reported separately for males and females in order to provide precise estimates. Difference in frequencies between males and females were computed using Chi-square Test. In order to present the prevalences of students’ physical and psychological health and wellbeing variables by university taking into account the varying male-to-female ratio of the samples at the different sites, we sex-adjusted the prevalences using direct standardization towards a male-to-female ratio of 30% to 70%.

In order to compare prevalences between study sites we used multivariate logistic regression to calculate Odds Ratios for each site while adjusting for sex. Deviation method was used as contrast method where each university as predictor variable is compared to the overall effect of the whole sample. For several variables, some of the response options were combined to satisfy the assumption of adequate cell size for regression analysis.

## 3. Results

Table 1 depicts some of the sample’s characteristics across the participating sites. More females where presented at most of the sites, probably due to the nature of the schools (e.g., Schools of Nursing, of Health Sciences, or of Health & Social Care, *etc.*) at each university where the data were collected. The differences in gender composition were less pronounced in the Gloucestershire sample. Participants had attended a wide variety of modules that contributed to several disciplines, although generally, health sciences were the main discipline at three universities, sport modules were only present at Gloucestershire, whilst the rest of the sample covered a range of disciplines. However it needs to be noted in the current multi-disciplinary trends in education that a given module’s content frequently contributes to more than one discipline. Higher proportions of Year 1 students were represented at 3 universities (Chester, Bath Spa, Swansea), while for the rest of the sample Year 2 participants contributed slightly more data, with the exception of Plymouth where it was the Year 3 students.

### 3.1. Socio-Demographic Characteristics of the Sample

Table 2 shows selected socio-demographic characteristics of the sample by gender. Across both genders, there were more of the younger students (age bracket 18–20 years), perhaps reflecting the nature of study in higher education institutions in the UK, where a substantial proportion of students are traditionally aged (‘fresh’ from high school). Females were more represented in the older age brackets (≥30 years, mature students). Males were more likely to be single, whilst higher proportions females were married and had children. Slightly more female students lived with their parents or with their partner, and fewer females lived with roommates when compared with male students.

Generally, female students were more likely to report that the income at their disposal was financially sufficient. Women felt that religion is very important in their lives, whilst more men somewhat or strongly disagreed to the statement.

### 3.2. Prevalence of Physical and Psychological Health Variables by Gender

Table 3 depicts the physical and psychological health profiles by gender. As regards physical health, males were more likely to rate their health better although females watched (kept an ‘eye’) their health more. During the 6 months prior to the survey, generally higher proportions of female students than males had consulted a medical practitioner, particularly at 3 or more occasions. In addition, women were more likely to report that in the past 12 months, they had been so ill that they had to stay in bed. Headaches were the most frequently reported health problems followed by back pain and neck or shoulder pain, where the rates of females complaining of such ailments were higher than of males.

As for psychological health, slightly more men than women felt that their quality of life was good. Although men reported that they usually had fewer persons to depend on for social support whenever they felt down, there were no gender differences in the satisfaction with the social support students received in such situations. The most frequent burdens encountered by the participants had to do with examinations, assignments and presentations issues, followed by financial concerns and other responsibilities that they had in addition to their study at university, where females were consistently more likely to report these burdens. Psychosomatic health problems were reported by both genders, although females experienced higher rates of such strains e.g., fatigue nervousness/anxiety and depressive mood.

### 3.3. Self-Reported Physical and Psychological/Mental Health and Wellbeing Variables across Participating Universities

Table 4 shows the comparison of sex-standardized rates of physical and psychological health variables for the whole sample and by university. The comparison revealed that some of the participating sites exhibited more favourable prevalences across many of the physical and psychological health variables under study. For instance students at site 3 generally reported a clustering of favourable levels of the variables under study: health problems/various strains (physical health) as well as burdens and psychosomatic problems/strains (psychological health) that were consistently lower than the sample’s average. In parallel, these students also reported social support and satisfaction with the support they received in such situations that were consistently higher than the sample’s average. Similar to this favourable pattern but to a lesser extent, students from site 7 also showed better rates than the sample’s average for four variables (staying in bed due to illness, burdens from studies in general and from exams, fatigue).

Conversely, compared to the sample’s averages, participants from site 6 exhibited a less favourable ‘overall situation’ across the physical and psychological health variables: a lower level of health awareness/consciousness (watch one’s health) and social support, in addition to higher rates of back pain, all types of burden, a higher prevalence of fatigue, and more consultations with a medical practitioner in the 6 months prior to the survey. The other participating universities did not exhibit such a clear pattern in any of the two directions, fairing well on some variables, and conversely doing less well on other variables when compared with the sample’s means.

## 4. Discussion

We investigated the perceived health status, in addition to physical and psychological health of students from seven universities in the England, Wales and Northern Ireland. Research that examines health and well being of university and college students has increased, because of the size and importance of this population [1,8,10,11,15,18,19,21]. Findings from the current study expand our awareness of the health needs and our appreciation of the health capacities of university students.

In relation to the first objective of the study, regarding the demographic findings, in our sample 27% of females and 11% of males had children, which was similar to levels that were reported in university students in Sweden where 31% and 17% females and males respectively had children [3]. As regards the financial situation of the students, about only half of our sample (59% females, 51% males) felt that the amount of money they have is either always or mostly sufficient. Although these percentages of UK students compared unfavourably with students in Spain or Germany who self-rated their income situation as sufficient (72% and 64% of the surveyed students respectively), the UK sample compared advantageously with Lithuanian students, where 38% reported sufficient income [8]. However, the rest of the UK sample who felt that the amount of money they have is either always or mostly insufficient could be disadvantaged: for instance, several studies have pointed out that healthy food consumption might be affected by the amount of money (financial resources) that an individual has at disposal [28–30]. Whilst research that relates financial situation and nutrition in university populations is generally scarce, for working adults in New Zealand, more money available for food could improve nutrition [28], as there were trends across socioeconomic status levels, with lower occupational classes, lower family income, and non-tertiary education groups having lower intakes of dietary fibre and calcium and higher intakes of dietary cholesterol [28]. Conversely, less money negatively influenced nutrition, where about one third of a sample of seniors in the USA either reported that household food supplies in the month prior to the survey did not last and there was not enough money to buy more; or could not afford to eat balanced meals; or that they had to cut the size of meals or skip meals in the past 12 months because there wasn’t enough money to buy food [31].

As regards the study’s second objective, we assessed the prevalences of many physical and psychological wellbeing variables. Self-rated health status can be reasonably used to compare health across different student populations [11]. In our sample, ≈90% ♀ and 88% ♂ students rated their general health as either good, very good or excellent. This was comparable to similar research of students in the USA (123 post secondary institutions) where 91% reported good, very good or excellent general health status [9].

In connection with health awareness (To what extent do you keep an eye on your health?), in our UK sample 85% ♀ and 81% ♂ reported that they watched their health to either some extent or very much. These levels were higher than in Spain or Germany (both ≈60%) but in the same range as in Lithuania (79%) [8]. Pertaining to health service use, about 65% and 48% of female and male UK students had seen a medical practitioner in the past 6 months, satisfactorily less than reported in university students in Spain (67%), Germany (82%), but in the same range as in Lithuania (57%) [8]. However it remains unclear, whether the lower use of health services in the UK students is due to actual lower needs or conversely, due to higher barriers of excess. Regarding the subjective health and pain complaints, strains and psychosomatic symptoms, 46% ♀ and 36% ♂ students in our sample suffered either sometimes or very often from back pain during the last 12 months. This is in agreement that back pain was the highest ranking complaint in the USA where 49% ♀ and 42% ♂ students reported it as a health problem experienced in the past school year [9], and matches findings from Spanish and German students who also reported more than 40% prevalence of back pain using the same rating scale as in our study [8]. However, in our sample, headaches ranked first for both genders (≈65% ♀ and 42% ♂), matching the levels of headache (52%) described elsewhere [3].

In connection with objective three, we assessed the prevalences of many variables of psychological wellbeing. In our sample, quality of life was rated quite well/very well by 64% of females and 68% of males respectively, where both levels were comparable with other studies undertaken in Denmark (67%) and the UK (65%) [19]. As for social support, about 8% ♀ and 11% ♂ of students in our sample had no social support or support of one person, which was nearly equivalent to levels reported in Spain (11.7%). However, the UK levels of lack of social support were higher than those reported in Germany (7%) but less than the levels in Lithuania (23%) [8]. The levels of perceived burdens were highest in relation to stress resulting from exams, assignments and presentations where 40% reported this stressor as either a strong or very strong burden. This suggested the high relevance of exams and assignments as sources of stress in relation to the physiological well-being of students. High burdens from study and work-related stressors have also been found in a similar study in students from England and Denmark, but the absolute rates are not directly comparable with our UK data due to the different cut-offs used [19]. As regards psychosomatic health problems/strains, whilst in our UK sample, depressed mood during the year preceding the survey was 31% ♀ and 23% ♂, in the USA, 20% ♀ and 14% ♂ reported depression as a health problem experienced in the past school year [9]. Indeed, depressive symptoms have been identified as a health problem among college/university students in many countries [32–39].

In relation to objective four, we compared the seven participating sites in relation to their students’ self-reported physical health and the mental/psychological wellbeing variables. A pattern of clustering of a ‘more favourable’ or ‘less favourable’ levels of the variables was observed across some sites. Whilst two sites showed levels that were more than the sample’s average in the favourable variables, and less than the sample’s average in the less favourable variables, another site exhibited the opposite pattern. However, generally most sites revealed mixed levels of favourable variables and of less favourable variables. It is difficult to postulate why such clustering patterns were observed. Such display of a collection (gathering) of ‘favourable’ or ‘less/un favourable’ health factors and practices could be related to a range of unique features that might characterize the university, its ‘environment’, its policies, and/or procedures for the selection of students and the resultant composition of the student population. Indeed a possible reason is that the differences could reflect the varying base student populations of the universities. It could also be related to the region where a university is located; or on a more general level, the country and its political and health stances. Moreover, one would normally expect many confounding factors (usually not measured) that would confound such complex and intricately associated constellations of relationships that are usually challenging to unpack, let alone attribute to certain aspects of the university, region, country or participating individuals. Elsewhere we have suggested the relationships of such findings with income, gender issues, political models, and social rights which could act as mediatory factors that might moderate attitudes [19]. On the other hand, at the individual person level, such clustering is understandable and conceivable, as perhaps habits and practices (whether healthy or less healthy) could cluster in certain individuals as shown by Allgöwer [40], bunch in certain groups, or crowd together in particular cohorts to collectively generate the greater picture. For instance, nearly 65% of women aged 18–22 enrolled full-time at an urban university in the USA had two or more unhealthy behaviours [5].

This study has limitations. It is a (descriptive) prevalence study and hence generalizations of the findings should exercise caution. Self reported data could be subject to sources of error e.g., recall bias, sociability and social desirability. In addition, for instance, health sciences disciplines and females were over-represented in this UK sample; and it is not clear how our sample universities compare with other universities in the UK. Hence we present our data categorised by gender and standardised for gender when undertaking comparisons across the participating sites. Although we standardized for gender, our male-to-female ratio might not be completely comparable to that of the UK as a whole. Some variables were assessed by single item measures due to respondent burden and the necessity of a general student health survey to be conducted within a short time in classes. This makes the use of measures with more items for each health factor unfeasible. Students were recruited during lessons, hence those not present in the class at the time of data collection were not included in the survey. Meanwhile, absence during lectures might be due to psychological and physical health problems. Despite our broadening of the data collection in an attempt that the selection of students in this study would be representative of their universities, even with our big sample and good response rates, our sample remains a convenience sample. Such convenience samples are not uncommon in student surveys: whether in Hong Kong [4] in the USA [41] or Australia [42]. In the USA, post secondary institutions (universities and colleges) self-selected themselves to participate in the American College Health Association National College Health Assessment survey [9]. The discussion of differences in health factors between the different universities is limited due to the fact that not all information on potentially differing conditions at the various sites (e.g., health-related environment and health services) could be collected and taken into account. Future research should attempt to address these limitations.

## 5. Conclusions

Overall the current study concludes that although health awareness was quite high and the use of the health services relatively was low in this sample of students from different universities in the UK, their level of health complaints and psychological problems/burdens fell within the same high range as observed in other student populations across Europe. The study also showed clustering effects of favourable as well as unfavourable health and wellbeing indicators among students from certain sites indicating the need for university-specific local health profiles as a valid basis for health promotion programmes at universities. Universities need to pay attention to the health and well-being needs of students.

## Figures and Tables

**Table 1.** Characteristics of the survey by participating sites.

**Variable**	**University**						
	**England**					**N. Ireland**	**Wales**

	Chester	Gloucestershire	Oxford Brookes	Plymouth	Bath Spa	Ulster	Swansea
	N = 993	N = 970	N = 208	N = 169	N = 485	N = 475	N = 406
**Gender**							
Female	86.9	56.4	89.2	63.9	77.4	91.8	92.2
Male	13.1	43.6	10.8	36.1	22.6	8.2	7.8
**Disciplines represented**							
Natural sciences	2.2	4.9	–	28.0	–	–	–
Social sciences	25.4	23.0	–	–	36.9	–	–
Sport sciences	0.0	31.0	–	–	–	–	–
Healthsciences	72.4	41.2	100	72.0	63.1	100	100
**Students’ year of study**							
Year 1 undergraduate	61.6	34.5	22.4	18.9	54.1	22.5	47.7
Year 2 undergraduate	22.3	36.6	48.3	34.9	23.4	44.2	23.6
Year 3 undergraduate	8.3	17.4	3.0	43.2	22.3	32.7	22.1
>Year 3 under-graduate or graduate/professional	7.8	11.5	26.4	3.0	3.0	0.6	6.5

All cells are column percentages.

**Table 2.** Socio-demographic characteristics of the sample by gender.

**Variable**	**Gender**		**p value**
	**Female** (n= 2,699)	**Male** (n = 765)	
**Age**			<0.001
18–20	42.5	50.7	
21–29	31.9	35.5	
≥30	25.5	13.8	
**Marital status**			<0.001
Single	56.7	68.8	
Married	18.7	8.5	
Other	24.7	22.7	
**Children** (Having children)	26.7	10.9	<0.001
**Living arrangements** (During semester)			
Living with parents	26.2	20.4	<0.001
Living alone	7.6	7.8	NS
Living with partner	28.5	15.2	<0.001
Living with room mate/s	35.4	56.1	<0.001
Other living arrangements	2.3	0.5	NS
**Finances** (The amount of money you have is)			
Always sufficient/Mostly sufficient	59.2	50.9	<0.001
**Importance of faith (**My religion is very important in my life)			<0.001
Strongly agree/Somewhat agree	26.9	20.9	
Neither agree nor disagree	27.4	23.4	
Somewhat disagree/Strongly disagree	46.0	55.7	

All cells are column percentages; P-values based on Chi Square statistics; NS: not significant.

**Table 3.** Physical and psychological health by gender.

**Variable**	**Gender^a^**		**P Value**
	**Female** (n= 2,699)	**Male** (n = 765)	
**PHYSICAL HEALTH**			
**General health**			0.001 ^b^
Excellent/Very good	46.4	52.1	
Good	43.2	35.8	
Fair/Poor	10.4	12.1	
**Watch one’s health** (To some extent/Very much)	84.6	80.7	0.01
**Seen medical practitioner in past 6 months**^a^ (Yes)	64.7	47.6	<0.001^b^
Among those			
1–2 times	70.3	76.9	
3–4 times	20.0	14.7	
≥5 times	9.7	8.4	
**During past year, been so ill that had to stay in bed** (Yes)	39.1	34.0	0.01
**Physical health problems/strains** (Sometimes/Very often)			
Headaches	64.5	42.3	<0.001
Back pain	45.9	35.9	<0.001
Neck or shoulder pain	41.6	32.4	<0.001

**PSYCHOLOGICAL HEALTH**			

**Quality of one’s life** (Quite well/Very well)	63.6	68.4	0.016
**Social support whenever you feel down**			0.004 ^b^
Low (None/1 person)	7.7	11.2	
Medium (2–3 persons)	27.2	23.7	
High (>3 persons)	65.1	65.2	
**Satisfied with support you get in such situations?**			
Very satisfied /Satisfied	70.2	71.7	0.430
**Burdens** (Very strongly/Strongly agree)			
Burdened overall	15.1	9.1	<0.001
Studies in general	24.3	16.9	<0.001
Exams, assignments, presentations	44.7	30.4	<0.001
Financial situation	30.5	28.9	0.414
Workload in addition to studying	32.3	20.0	<0.001
Lack of time for studies	27.7	16.9	<0.001
**Psychosomatic health problems/strains** (Sometimes/Very often)			
Fatigue	65.3	46.6	<0.001
Nervousness/anxiety	47.4	28.6	<0.001
Depressive mood	30.5	22.5	0.130

All cells are column percentages;

a Does not include seeing a dentist;

b P-value refers to Chi-square test over all response categories.

**Table 4.** Sex-standardized^†^ rates of physical and psychological health indicators for whole sample and by university.

**Variable**	**Whole sample**	**Site 1**	**Site 2**	**Site 3**	**Site 4**	**Site 5**	**Site 6**	**Site 7**	**P value^a^**
**PHYSICAL HEALTH**									

**General health** (Excellent/Very good)	48.1	45.2	50.5	54.0	53.7	42.6 *	44.9	49.8	0.044
**Watch one’s health** (To some extent/Very much)	83.4	76.9	79.4	89.6	87.9	80.1	78.1 **	86.6	0.017
Seen medical practitioner in past 6 months * (Yes)	59.6	54.7	58.0	64.5	64.3	68.3 **	66.5 **	70.0	<0.001
**During past year, been so ill that had to stay in bed** (Yes)	37.6	34.3	37.2	36.7	42.0	49.4 ***	35.0	32.7 *	<0.001
**Health problems/various strains** (Sometimes/Very often)									
Headaches	57.8	59.5	54.4 *	51.9	59.3	62.9	63.3	58.6	0.039
Back pain	42.7	41.6	42.9	49.1 *	40.8	39.6	50.2 *	42.2	0.020
Neck or shoulder pain	53.3	37.9	38.1	46.6	40.4	40.8	41.0	37.8	0.478

**PSYCHOLOGICAL HEALTH**									

**Quality of one’s life** (Quite well/Very well)	65.0	60.7	67.5	70.0	61.0	66.1	60.7	70.1	0.010
**Social support whenever you feel down** (High ≥ 3 persons)	65.1	66.6	67.6 *	54.7	65.5	66.5	58.9 *	62.5	0.029
**Satisfied with support you get in such situations?**									
Very satisfied/Satisfied	70.5	67.9	73.1 *	69.2	77.4	72.3	66.7	71.9	0.068
**Burdens** (Strongly/Very strongly)									
Overall burdened	12.9	16.9	6.4 ***	21.0	12.1	15.1	14.4	12.2	<0.001
Studies in general	22.1	20.8	15.8 ***	29.3	33.1**	21.0	31.3 ***	15.1 **	<0.001
Exams, assignments and presentations	40.4	41.5	33.8 **	36.0*	43.7	41.2	48.7 ***	32.8 **	0.020
Financial situation	30.0	28.1	23.4 ***	32.6	34.1	33.9	39.2 **	30.7	<0.001
Workload in addition to studying	28.6	30.4	21.1 ***	43.7**	24.9	23.2 **	41.0 ***	26.0	0.007
Lack of time for studies	24.5	29.0	16.3 ***	37.0**	28.0	17.8 ***	30.6 **	20.3	<0.001
**Psychosomatic problems/strains** (Sometimes/Very often)									
Fatigue	59.7	62.4	54.6 ***	69.5 *	60.6	63.1	66.4	53.4 **	<0.001
Nervousness/anxiety	41.8	40.5	37.9 **	34.9	42.6	48.3 **	56.9 ***	38.4	<0.001
Depressive mood	28.1	30.5	26.3 **	24.4 *	32.6	35.6	38.5 **	29.0	<0.001

† Male-to-female ratio of 30:70, all university sites are anonymous for confidentiality; all cells are sex-standardised percentages of the given variable/categories (row) listed for the different samples (columns), values in bold indicate statistical significance;

a *p*-values for an effect across the participating universities based on logistic regression models adjusted for sex; Significance levels indicate differences between each university and the whole sample, *i.e.*, each university compared to the overall rate, where

* *p* < 0.05,

** *p* < 0.01, and

*** *p* < 0.001.
